# Chronic Pyæmia of Dental Origin

**Published:** 1887-10

**Authors:** R. J. Porre

**Affiliations:** Cincinnati, Ohio, U. S. A.


### Chronic Pyæmia of Dental Origin.

BY R. J. PORRE, D.D.S., Cincinnati, Ohio, U. S. A.

Read before the Section on Dental and Oral Surgery Ninth International Medical Congress.

A Synopsis of a Case of Peculiar, Intense, and Long-continued Blood Poisoning. —Complications Resembling Constitutional Syphilis—Under Treatment by Thirty-five Different Surgeons During a Period of Thirty Years.

In November, 1882, the writer read before the class of the Ohio Medical College, by request of Prof. W. W. Dawson, a paper giving a description of a case of pyæmic suffering extending over a space of thirty years, and of a character so protean and altogether extraordinary as to baffle the diagnostic and therapeutic skill of many of our best surgeons. Patient and careful investigation, from what may be termed a dentist's standpoint, disclosed an abscess caused by a concealed suppurating wisdom tooth. After removal of this tooth and proper treatment the patient's health was speedily restored. This paper was subsequently published in the Cincinnati *Lancet and Clinic*, November 25, 1882. It was extensively copied into American and foreign medical journals, and excited considerable interest, eliciting comments both favorable and, to some extent, unfavorable; a majority congratulating the author upon having discovered a heretofore unsuspected cause for many obscure and grave disorders, and others, while admitting the general facts and conclusions, affecting to believe that the varied terrors and protean phases of the symptoms described were perhaps exaggerations on the part of an extremely nervous patient or a highly imaginative dentist.

No one could possibly be more anxious than the "dentist" to be able to verify the accuracy of the symptomatology and correctness of the conclusions.

The latter may certainly be disposed of briefly with the remark that whatever may have been the cause of the long train of varied tissue and functional disorders, they all disappeared after the removal of the supposed offending tooth and obliteration of the local abscess.

It may be of interest to state that up to the present time the patient enjoys absolute immunity from the previous complications.

In regard to the pyæmic symptomatology suggested by the history of the case, the author is now able to demonstrate, as the result of four years' additional experience, a complete medical syllogism, from verified premises to an assured conclusion.

In order to do this it will be necessary to refer to the remarkable train of symptoms in that case, compared with the admitted results of pyæmia by surgical authorities, and if possible verify the conclusions by the testimony of other and similar cases.

It will be remembered by those who read the details of the case named that its startling features consisted in the vast variety and persistent development of lesions in so many tissues, apparently so very discrepant with any theory of dental origin. The patient, it may be well to state, inherited a powerful constitution, of active habits from his youth, an athlete, possessed an iron will and an irrepressible spirit, all of which sustained him in his struggle with this hydra-headed monster of diseases. He remembered that thirty years before he had a facial swelling, which had subsided after a week's diet and treatment. Since that time he had never been quite free from neuralgia and pains in some other parts of his organism. He had been the victim of constantly recurring furuncles and eruptions in various parts of the body, which would often for months become running abscesses. He experienced burning and itching eruptions of the hands and feet, which would finally change to stubborn ulcerations.

His throat was subject to frequent and painful inflammations. His bowels were either stubbornly constipated or exhaustingly loose. He suffered from frequent rigors and febrile attacks of varying intensity, profuse night sweats, retention of urine, serious constriction of the bowels and urethra. Lancinating pains like electric shocks darted from maxilla of the right side to diaphragm, bowels, bladder, limbs, hands, and feet, or to whatever part that was locally affected at the time. It was this latter peculiarity, together with the discovery of a little pus exuding from the locality of the wisdom tooth, that lead to a finally correct diagnosis of his case. He had been treated in turn for nearly every local lesion, and for over a score of years for syphilis. It is not necessary to repeat the treatment adopted and final relief of this patient. It has been given in detail.

The important point is to establish the relations of cause and effect between these apparently trivial local abscesses from diseased teeth and the numerous and diversified symptoms of pyæmia.

Surgically considered pyæmia expresses that class of morbid effects produced by animal products upon the system.

It will be interesting to observe the curious resemblance between the diversified effects of the almost innumerable phenomena produced by the distribution of the morbific matter among different tissues under varying circumstances and the cases we have to offer for investigation. The conditions may be primary, that is, directly produced by the poisonous matter, or secondary, when complications occur from the systemic infection.

They may be general and rapid, as the virus is absorbed into veins and lymphatics, attended by fever, rapid pulse, heat, vertigo, rigors, perspirations, etc., modified in intensity by the extent of the abscesses or reservoir from which the supply of the virus is drawn. The general demoralization of the system in such cases is indicated by changes in the ulcer or source of inoculation.

If from a wound granulation is arrested, and sometimes the cicatrix is broken down, the margins become dusky and indurated, the integument becomes distended with serous fluid, a sanious discharge takes the place of pus. Purpura and ecchymoses occur in different parts of the body as the morbid effects disseminate, the patient becomes anxious and delirous until prostration, sinking, and death close the scene. Or if the morbific supply is arrested before the utter demoralization of the vital functions, typhoid symptoms supervene, the system labors to eliminate the poison by fluxes of various kinds, and, if successful, convalescence is slow and feeble.

Or the effects may be modified by various circumstances—disease, age, temperament, state of health, habits, and idiosyncrasies. For example, the influence of scarlatina, diptheria, phthisis, cancer, shock, wounds, etc., aggravate and increase the action of the virus.

The symptoms vary also with each new organ affected, and nothing is more usual amid the general symptoms of depression and prostration, attended with anorexia, sweetish taste in the mouth, distaste for food, sallow or tawny skin, to note discharges from the nose, ears, or uterus, or effusions into serous cavities, and cellular tissues, boils,abscesses,etc.,in parts distant from each other.

The gravity of all such complications being determined by the quantity of infection and resistance of the vital forces to its influence.

Now if all of these pathological conditions may follow some mysterious local change or decomposition of the animal substance, perhaps the case cited may not seem so improbable or exaggerated.

It is an axiom in mechanics that the strength of a piece of machinery is only equal to its weakest place, and any intelligent surgeon will tell you that it is the only explanation he is able sometimes to give to his curious patient of the capricious action of general diseases upon certain local organs.

Suppose a wandering pus corpuscle, or several of them, should find their way into the circulation from some suppurating tissue, a vigorous vitality would be able to eliminate and dispose of them. Or the globules might in succession pass the lungs, liver, spleen, and kidneys, and finally get entangled in some debilitated capillary of the skin, and so soon as its progress is arrested its work of degeneration begins, and a local abscess or furuncle is the result. But the keen and tireless investigations of science have demonstrated that the pus globule, while in the exercise of its wonderful task in the process of waste and repair, is not the dangerous enemy it has been represented, but must itself be demoralized, if we may use such a word, to signify the condition of decomposition and chemical change produced in animal substances by various causes, among which may be named micro-organisms and certain chemical products of the putrefaction of proteid substances, alkaloidal in their character, and of peculiarly virulent and destructive nature; to which Gauthier, in 1870, gave the name of ptomaines, to distinguish them from certain alkaloid principles found normally in animal excretions, as urine, saliva, etc., to which the name of leucomaines is given.

Gauthier also demonstrated the presence in the secretions, even in health, of certain nitrogenous extractive compounds of a highly toxic nature, to which the bites of animals often owe their poisonous character.

It is not necessary at this time to discuss the various theories of chemical, microbic, or fermentative origin of these insidious morbid elements in the system, since we are too frequently rendered painfully aware that life is a continued series of deaths and resurrections, and while we carry about with us the elements of death, and the corpse of dead protoplasm in every tissue, we retain life and resist the infection from morbid chemical changes and a thousand inimical inferior organisms only by our superior vital powers, whereby we eliminate the poisonous substances and promote their destruction by oxygen.

Our present affair is with known results rather than with the discussion of theories concerning the origin and influence of microbes or the ptomaines and leucomaines of dead or living tissue upon the body. The remarkable analogy between the admitted pathology of known pyæmic diseases and the train of symptoms presented in the case of dental lesions already described may now be so fully confirmed by subsequent experience that the relation of cause and effect is no longer in doubt; as in all toxic diseases, when the system becomes affected by depraved secretions, as phthisis, tonsillitis, and diptheria, ulcerations of the ears and throat, and abscess of the viscera, etc. It is well understood that the gravity of the effect is determined by the strength of the vital forces to eliminate the infection, and the rule obtains in the class of disorders produced by diseased and carious teeth, the morbid results, as from other forms of vitiation being proportioned to the magnitude of the cause, the idiosyncrasy of the patient, and the amount of vital resistance.

It must also be remembered that the oral cavity is subject to other diseases whose morbid secretions are capable of vitiating healthy action of other functions, among which may be named scurvy, of whose terrors it is not necessary to remind the reader. And when the disease is cured not unfrequently the consequences, as diseased gums, loosened teeth, etc., become prolific sources of future general vitiation.

Pyorrhea alveolaris is another local lesion from which the gravest pyæmic disorders may be feared, all the more so as diagnosis is often obscure and the mischief is quietly going on without the cause being suspected. The disease consists in inflammation, ulceration, and caries of the investments of the teeth, and often the discharge of pus is phenomenal in quantity. The gums waste less at their borders than at the margins of the alveola. The deposits around the neck and roots of the teeth, in some cases, are unlike salivary calculi, being dark, hard, and so adherent as to almost defy removal. This deposit is regarded as produced in some manner from elements of the blood ; therefore it has been named calculus sanguinarius.

The etiology of this disease has not been satisfactorily settled as to whether it is of local or constitutional origin.

Another serious disorder of this class is alveolar abscess, which, although most frequently caused by carious teeth, producing periosteal inflammation, is often variable in its forms of development. A suppuration may begin around the root, absorbing the bony walls; pus burrows its way sometimes along the tooth to the edge of the gums, generally through the gums opposite the apex of the root, not unfrequently opening on the cheek, chin, jaw, and points remote from the place of beginning.

Often the antrum is implicated, and a series of painful and grave results occur with which dentists and surgeons are familiar.

The process and effects of caries is both suggestive and interesting in this connection. The periosteum thickens and becomes highly vascular, beside the ovoid tissue cells rows of spindle-shaped cells, forming bundles of wavy connective tissue, appear; small nerves and vessels in the vicinity atrophy and large vessels expand, tissue undergoes a retrograde metamorphosis, fatty granules appear in and between the cells of protoplasm, and germs of proliferation are excited. The root is absorbed, the plastic exudation elevates and loosens the tooth. Suppuration begins in the lymph around the tooth and in the cavity formed by absorption, and is often very rapid and always painful.

Although only a toothache, readily cured by a pair of forceps, the carious process may extend by means of the absorbents and lymphatics, which convey the infectious matter to other and distant organs, and by a little stream, whether of decomposed pus or ptomaines, too small to destroy life outright by pyæmia, but enough to vitiate the health of the whole body, is able to produce a degree of physical suffering wholly incommensurate with its inconsiderable origin.

The symtomatology of pyæmia and methods of dental disease have been given somewhat in detail in order to compare with the following cases now offered in corroboration of the theory of pyæmic poisoning suggested by the history of the one described in 1882.

---

[Case No. 1.]

### CELLULAR INFLAMMATION OF NECK, SIMILAR TO MALARIAL FEVER.

In July, 1881, Mr. K. was sent to me for diagnosis and treatment by Prof. W. W. Dawson, of the Ohio Medical College.

The patient was thirty years of age and of good physical history and habits. He was suffering under a fourth repetition of an excessive swelling of the left side of the face, extending downward quite to the clavicle. The first attack had occurred a year before, and he had been continuously under treatment of some kind, and suffering great local and general distress of body and mind under the conviction that the disease was malignant. He came to Prof. D., who opened the buccal wall into a cyst, which discharged a teacupful of yellowish sanious fluid at intervals during a week or so, gradually diminishing in quantity and the symptoms subsiding into an apparently normal condition. As symptoms of malignant or constitutional disease were absent, and as effects do not exist without causes, it became necessary to seek for some occult source of irritation sufficient to account for such results. The mouth was carefully explored. The first and second bicuspids on that side were missing. The three molars exhibited no tenderness, and had but slight superficial caries on their grinding surfaces.

No evidence of roots or appearance of inflammation in the gums were visible on that side.

With less experience, or less firmly established convictions of the dental origin of such disorders, a correct diagnosis seemed like a forlorn hope. But in accordance with a custom born of experience not to rely too much upon appearances, I began a systematic search for concealed roots, and finally found the remains of the first bicuspid. It was not easy to determine whether doctor or patient was most pleased when the probability dawned upon them that the cause of the long suffering had been found. Upon removal of the root the stench was intolerable, although the exudation was but slight.

A free opening with the dental engine revealed the sinus impacted with a cheesy deposit. This was broken up and the cavity washed out with an antiseptic lotion of following formula: Carbolic acid, oil eucalyptus, chlor. zinc., alcohol; aq. distil.

This treatment was continued daily for ten days, and a complete cure effected, and the patient relegated to the care of his physician to recuperate his general health, which had been much deteriorated by absorption of the vitiated secretions, combined with local pain and a wearing dread of malignant disease.

It is not difficult in this case to trace the array of pyæmic symptoms directly to a dental origin. The obscurity and apparent unimportance of which had lead to the gravest misapprehension in regard to both diagnosis and treatment.

---

[Case No. 2.]

### SERIOUS INVOLVMENT OF THE EYE—GREAT GENERAL PROSTRATION.

In May, 1885, Dr. McDermott brought to me for diagnosis and treatment Mr. S., a young merchant from Kentucky, 28 years of age, who had consulted Dr. McDermott for severe pains of the right side of the face and eye. Suspecting some obscure dental origin and observing symptoms of oral abscess, the doctor referred the case to me. The patient was of good physical ancestry, and with the exception of an interregnum about the age of 20, had enjoyed excellent health until 18 months before, when it began to fail. He suffered from loss of appetite, indigestion, and unaccountable mental depression and general nervous prostration, for which his physician had recommended sea air and bathing. The fatigue of the journey to New York had so exhausted him that he returned with some difficulty, and only after a severe illness of two months and skillful tonic treatment was he sufficiently recuperated to resume business. For a time his general health greatly improved, with the exception of occasional severe facial pains, involving the right eye, for which he had finally sought the advice of an oculist.

Now certainly no diagnostic symptoms could have been more obscure, and his medical attendants would most naturally be disposed to seek for causes in functional disorders of the digestive system. Yet the predominance of a typhoid tone, and the persistent neuralgic symptoms indicated plainly the presence of some vitiated secretion as a far more probable origin of the vital deterioration.

I found an abscess occupying the entire right half of the roof of the mouth.

Seeking to detect its origin, I suspected a right upper bicuspid, the plug of which was leaking, but upon investigation found the pulp alive, and I capped and refilled the cavity.

Percussion revealed no tenderness in any tooth. Fluctuation in the abscess was unmistakable, but the outlet of the secretion under quite hard pressure was obscure, but upon patient and careful investigation I finally discovered a detachment at the margin of the gum back of the right upper cuspid, presenting no evidence of disease, but through which, by hard pressure, I obtained a small exudation of sanious fluid.

I made a free opening at this point, and was surprised at the quantity of purulent matter which was forced out by a pumping motion with pressure upon the sac. I washed the sinus out freely with the " antiseptic lotion," and put in a drainage tube.

Further investigation for cause discovered caries in the alveolus, at the base of the cuspid mentioned—no caries in the tooth. The secretion had been retained until the soft tissues lining the roof of the mouth became detached and distended, and every morning the patient could by hard pressure force out small quantities through the valve-like margin of the gum. The free opening I made facilitated the complete evacuation of the sac and sinuses, and enabled me to treat the abscess, and in a short time I turned the patient over to his medical advisers, and as I have not since heard of him I presume that the cure was complete.

---

[Case No. 3.]

### GENERAL PROSTRATION WITH TRAUMATIC LESION OF THE TONGUE.

I extract from my notes the following pronounced case of pyæmia from dental origin. The patient was one of Dr. Thrasher's, who had diagnosed well marked symptoms of pyæmia, and requested me to call with him to examine the mouth for cause. The patient, Mr. W., aged 68, had a good physical history. For some years had been suffering from a general failure in health. A few months before my visit, as Dr. Thrasher had obtained the history of the case, and he found the patient's condition was of a low typhoid character, great mental depression, cough and expectoration, indurated patches detected over lower lobe of the left lung, tongue coated and aphthous, anorexia, irregular bowels, and pulse quick and feeble. Under a tonic treatment he would react for a time and then relapse. We found his mouth in bad condition, teeth covered with tartar, and gums ulcerating. Two of his few remaining teeth were ulcerated and discharging septic fluid. I desire at this point to call especial attention to a method of propagating infection that is perhaps too frequently overlooked. In this case one of the ulcerated teeth, reeking with ptomaines and purulent virus, was a right lower molar, which, inclining inward and worn to a sharp edge, continually cut into the tongue, and thus actually distributed the virus by inoculation. The wound in the tongue had formed a sinus, running obliquely backward toward the base one and one-fourth of an inch. The two ulcerated teeth were extracted and the usual antiseptic lotion applied, and Dr. Thrasher informs me that the patient's health is now better than for years.

[Case No. 4.]

Mrs. P., 32 years old, and of good physical history, under treatment for pyæmia by Dr. Coffman, is another case in illustration of what the foreign journals are pleased to call "my theory of dental pyæmia."

This lady had suffered from the entire list of protean tortures for many years, and had been treated for nearly every form of functional disorder, and of course without success.

I will mention but one peculiarity in her case, which is particularly significant, as indicating the capricious tendency of pyæmic poison to affect various tissues and to develop in inexplicable ways and places.

This lady was affected with painful furuncles of the scalp, and after they were opened and contents discharged the cysts would secrete a purulent sanious fluid, which at times burrowed under nearly the entire scalp in sinuses that anastomosed, and required frequent tapping to evacuate the fluid. A complete cure followed the extraction of ten diseased teeth and roots, treating and filling several others, and cleaning a large amount of tartar from the remaining teeth.

---

[Case No. 5.]

INFLAMMATION OF THE THROAT.

Another of the varied expressions of dental pyæmia I find in the case of a lady brought to me by Dr. A. for consultation and treatment. This patient had been treated by several prominent physicians during a series of years for a disease of the throat, involving at times the pharynx and fauces, inflicting very serious local distress and a general deterioration of health, both mental and physical. The doctor recognized the effects of some septic infection, and upon consultation after extracting several ulcerated teeth, and especially the root of a left lower wisdom tooth, the lady's health rapidly improved under the most simple remedies. Her throat disease disappeared, as the cause was evidently the ulcerating third molar root.

[Case No. 6.]

COMPLICATION OF FACIAL PARALYSIS.

Miss A. C. aged 20, living in a neighboring city, about three years ago had a large cavity filled in the left lower second molar. In a few days the tooth became painful. She called on her dentist for relief, but he persuaded her that it was only an attack of neuralgia and would be temporary. For two years, with only short intervals, she continued a sufferer. At times the pain was intensely severe, involving the entire side of her face and head, until finally her naturally strong and robust constitution gave way under the serious complications, when she called a physician, Dr. T. This was last November. The doctor, in diagnosing her case, could discover no cause for her condition, if it was not produced by her teeth, and advised that as soon as she was able to visit her dentist for treatment. But her case became rapidly more serious from day to day; the pain intensified and the swelling increased until the left eye was involved and protruded to a frightful degree and almost unendurably painful. This condition continued for about three weeks, when symptoms of paralysis were manifested by falling of the lip. Although every thing was done locally and generally to avert the attack, it finally implicated the entire side of the face and became a permanent complication to her already distressing case. After the paralysis of the face became complete, which was in a few days, the pain in the tooth and side of the head suddenly subsided. During four or five months she became greatly reduced in flesh—the shadow of her former self. Generally, she experienced the usual serious functional derangements clearly pathognomic of pyæmia unnecessary to particularize. She began slowly to regain strength finally under the effect of strong tonics, and as soon as she was able was brought to my office, when I got the history of her case. Upon examining the tooth designated I found it the only one tender under percussion on that side. The gums showed but slight evidence of inflammation or swelling around it. Her enfeebled and nervous condition forbade extracting the tooth, so I removed the filling and a liberal exudation, for so slight external evidence, of an odor of no uncertain character followed. The fact should be noted that this was a plain case of chemical decomposition and putrefaction, as there was no opening to the sac, and the secretion was evidently as toxic in its action by absorption as that from an open abscess or ulcer. The tooth was thoroughly syringed with warm water and dressed with the following lotion, and patient dismissed: Chloral, gum camphor, carbolic acid, grs. xxx; ol eucalyptus ℈; acid salacylic, grs. x; alcohol, ℥j; aqua distil., ℥ss.

Upon her return in three days the improvement was astonishing. She reported that she had not felt so well in three years, that all the uncomfortable symptoms were rapidly subsiding, that her appetite had improved, and that she slept soundly and refreshingly, etc. She calls at intervals of three or four days to have the tooth treated, and the metamorphosis is positively phenomenal. She goes out in the open air unrestricted day or night, which she did not nor dared not do before the tooth was treated. And the promise is now, since a month has elapsed, that the tooth will be saved, the paralysis dissipated, and that at an early period she will realize a full and complete restoration of her former excellent health.

---

[Case No. 7.]

As an example of the surprising rapid and complete recovery of persons aflicted with pyæmia from oral lesions after proper attention, I will mention a case which is representative of many.

Mr. W., aged 64, had enjoyed the best of health until four or five years previous, during which time he had received medical attention, but to no avail—evidently for reason of failure to discover the cause of his disorders. He became the patient of Dr. Thrasher. The doctor, after obtaining the history of his case, examined for the cause of his long train of ills, recognizing plainly aggravating symptoms of pyæmia, and, as in instances before, he examined the mouth for cause. Finding several ulcerating teeth and roots and badly diseased gums, he brought the patient to my office for the operation very plainly indicated. Ether was administered, and seventeen teeth and roots were extracted.

In ten days he had gained in weight six pounds, indisputably proving that the septic poison generated by the diseased condition of his teeth and mouth was the cause of his seriously disordered system, as his rapid and complete restoration to health after the cause was removed attested.

---

[Case No. 8.]

EXTENSIVE CARIES AND NECROSES OF THE ALVEOLUS.

In 1885 Mr. B., aged 64, history good, called for consultation. I found the left superior maxilla from the lateral incisor to the second bicuspid and to the base of the sinus, with almost all of the alveolus investing the teeth, involved in caries. Over the cuspid and the space of the first bicuspid, as it was gone, there was an opening through the gums into a cavity in the alveolus, caused by necrotic disintegration, that would receive a filbert. Three or four years before he experienced an abscess over the cuspid which spontaneously opened, and had continued to discharge a sanious secretion afterwards. He first applied for, and had been under, treatment more especially to be relieved from the unpleasantness and offensiveness of the excessive discharge from the fistulous opening, as there was no pain. Having been under local treatment a year, with the promise of a cure, and no change, he began to think his case a serious one and decided to seek other advice. The treatment was plainly indicated, namely, the extraction of the three teeth mentioned. This was accordingly done, and the carious bone cut away and washed out with warm water and the septic lotion, and the patient was requested to call daily. It was apparent after ten days' treatment that a condition still prevailed that was antagonistic to the reconstruction of bone. Examination with the probe revealed extended ramifications of carious bone. So after drying the cavity with absorbent cotton, the nozzle of a syringe charged with a drachm of sulphuric acid was introduced into each of the several ramifications and a few drops discharged, and also a few drops were deposited upon surfaces of bone still exposed on the walls of the cavity. (The acid was successfully used in the first case mentioned in this paper for similar conditions.)

At the end of a week there was a decidedly favorable change. The walls of the lesion became rapidly covered with granulations. With the exception of two or three applications each, of two deep-seated points of caries afterwards with the acid, and the daily use of the lotion as usual, the reconstruction of bone was complete in thirty days. The health of the patient, which was deteriorating with marked symptoms of pyæmia, was speedily restored to its former excellent standard, and is still unusually good for a gentleman of his age.

---

[Case No. 9.]

GRAVE GENERAL DEBILITY WITH SERIOUS COMPLICATION OF THE UTERUS.

Mrs. G., widow, aged 35, of good personal and family history, had been an invalid for years, and for a year or two before I saw her had been under the care of a gynecologist. Circumstances required that she should give daily personal attention to household duties. Consequently, at times her sufferings were severe and exhausting, until finally compelled to surrender to her couch and receive special treatment for a week or two, when she would resume the dragging and, to her, hopeless life of agony. She called to consult about the painful and unpleasant condition of her teeth and mouth. Upon examination there was but one course suggested, namely, the extraction of fifteen teeth and roots, as they were hopelessly decayed, and the gums and alveolus were seriously involved in ulceration and caries, with an excessive secretion and discharge of septic fluid. Being abnormally nervous, she deferred the operation for some two or three months, when she finally returned to obtain the relief confidently promised, as she received no permanent benefit otherwise. An obtundant lotion was applied to the gums, and the operation was at once successfully completed. The nervous shock was singularly light. After the lapse of a week, the improvement was astonishingly rapid, and in the course of two months her recovery was complete, declaring herself in perfect health, and having regained to the maximum her lost weight. The uterus is perhaps the most sympathetic organ to septic causes of the entire organism, let the generation of septic matter originate or be produced by whatever source or cause. An involvment, it may be observed, either in ulceration, congestion and falling, or what not, adds as a secondary cause of aggravation to the constitutional derangement. The above case is representative of many that have been noted by the writer, and is worthy of especial attention by gynecologists. Where traumatic causes are not present an examination into the condition of the oral cavity will perhaps reveal in a majority of cases the source of vitiation and cause of organic lesion.

A few words in behalf our little ones in this connection can not but be appropriate, especially for the reason that the fact may not be generally known that the temporary teeth of a majority of children, perhaps, from causes unnecessary for the purpose of this paper to discuss, between the ages of three and twelve years, are more or less extensively affected with caries. Therefore, as there is rarely a limit to its ravages, distressing toothache prevails, the nerves devitalize, then follows in time the death of the teeth, when nature, through her unvarying methods, proceeds by inflammation and suppuration to dispose of them; and if they are neglected and allowed to remain not only interfere with the eruption of the permanent teeth, but will inevitably afflict the unfortunate victim with this most insidious and relentless of diseases.

It may be noted that when this condition prevails the injurious results, as with an adult, so with a child, are in proportion to the amount of septic poison diffused and the vital strength to eliminate it. Some children are peculiarly susceptible to toxic effects of all kinds. If there are opportunities they are victims to all of the contagious diseases incident to childhood. So one collapsed abscess even, secreting and exuding purulent fluid, which in fact would be sufficient, and is not uncommon, to vitiate the organism of an adult; consequently, if the enfeebled child having not only one diseased tooth, but unfortunately a generally disorganized mouth, not only suffers grave pyæmic derangements, but should it while thus afflicted be attacked with diptheria or scarlatina, or any serious epidemic disease, its chances of recovery would be very much lessened. These are facts certainly worthy of attention, and can not but impress those having the care of children, either professionally or otherwise, as of the greatest importance. The sensitiveness of some children to the effects of apparently the most trivial lesions is as distressing as it is a surprising fact. What physician has not sat by the couch of a little sufferer, in convulsions perhaps, or in consuming fever, utterly helpless to administer relief, and seen it pant its life away from no other cause than irritation produced by an erupting tooth. In diagnosing the cases of children, if the foregoing are facts, which are confidently submitted to the observing for corroboration, inspection of the mouth, therefore, will disclose in very many cases conditions requiring immediate attention.

There is a class of cases with which observing dentists are becoming more familiar, where you have only to repeat some of the varied manifestations of pyæmia to describe your patient's case. For example, a lady calls for consultation in regard to diseased and painful teeth. She has a pallid and clayey color and is extremely nervous and weak. After careful examination of her mouth, and you find one or more ulcerated teeth, you may say confidently: "You must have suffered greatly, you have no appetite, your digestion is very bad, you spit up your food, your bowels are constipated, your nervous prostration is sometimes so extreme that you suffer from night sweats, rigors, and fever, your uterine troubles have become chronic and serious, you have darting pains through various parts of the body. Often you can not sleep from unrest and nervousness; you have had, perhaps, paroxysms of hysteria; sometimes you have burning of the hands and feet; your temper is variable, and makes you at times very unhappy and depressed, etc."

The chances are ten to one that she will assent to all you have said, expressing the greatest astonishment that you have read her case so well.

Her happiness and confidence in you are rendered complete, when after the removal of the exciting cause and simple treatment for a few days she finds her aches and pains all gone and her health returning.

Physicians are awakening to the importance of exploring the mouth in all cases of pyæmic and neuralgic symptoms of obscure origin, and, having long regarded all diseases of the teeth as the special province of the dentist, are now bringing ten such cases to him for diagnosis and consultation where one was brought before.

My own experience is to the effect that but few cases afford greater satisfaction to physician, dentist, and patient than such as I have described, selected from notes of many similar ones. I am also quite certain now of the correctness of my conclusions, as expressed in 1882, in regard to the pyæmic results of obscure dental lesions.

I leave the facts and theories with my dental brethren and the medical profession with entire confidence.

